# One-year outcomes of combined phacoemulsification and goniosynechialysis in primary angle-closure glaucoma with cataracts: a multicenter prospective study

**DOI:** 10.1186/s40662-026-00507-y

**Published:** 2026-08-20

**Authors:** Kun Xiong, Wuliang Li, Qingyi Liu, Shaodan Zhang, Guoxing Li, Huiyan Mao, Qiangjie Huang, Qi’ao Zhang, Rongrong Le, Yanqian Xie, JingJing Zuo, Bo Yu, Yachen Wang, Xiaoping Xu, Xiaoli Xing, Lijun Zhao, Lu Yang, Yuanbo Liang, Ningli Wang

**Affiliations:** 1 Jiangxi Clinical Research Center for Ophthalmic Disease, The Affiliated Eye Hospital, Jiangxi Medical College, Nanchang University, Nanchang, 330006 China; 2 National Clinical Research Center for Ocular Diseases, Eye Hospital, Wenzhou Medical University, No. 270, Xue Yuan West Road, Wenzhou, 325027 Zhejiang China; 3 Ningbo Key Laboratory of Medical Research On Blinding Eye Diseases, Ningbo Eye Institute, Ningbo Eye Hospital, Wenzhou Medical University, Ningbo, 315040 China; 4 Aier Eye Hospital of Wuhan University, Wuhan, China; 5 Tianjin International Joint Research and Development Centre of Ophthalmology and Vision Science, Eye Institute and School of Optometry, Tianjin Medical University Eye Hospital, Tianjin, China; 6 Department of Ophthalmology, The Third People’s Hospital of Dalian University of Technology, Dalian, China; 7 Department of Ophthalmology, Beijing Tongren Hospital, Capital Medical University, Beijing, China

**Keywords:** Primary angle-closure glaucoma, Phacoemulsification, Goniosynechialysis, Multicenter prospective study

## Abstract

**Background:**

This study aimed to observe the safety and effectiveness of combined phacoemulsification and goniosynechialysis (PEI-GSL) in patients with primary angle-closure glaucoma (PACG) and coexisting cataracts.

**Methods:**

This multicenter prospective study enrolled 289 consecutive patients with PACG and cataracts who were treated using PEI-GSL between November 1, 2022 and March 12, 2024. All patients underwent comprehensive ocular evaluations at baseline, at 1 week, and at 1, 3, 6, and 12 months postoperatively. Complete success was defined as achieving an intraocular pressure (IOP) of 6–18 mmHg without antiglaucoma medications or reoperation. Qualified success was similarly defined but allowed antiglaucoma medications.

**Results:**

Among patients with PACG, 237 (82.0%) of 289 completed a 12-month follow-up. The baseline IOP decreased from 22.9 ± 10.2 to 14.0 ± 3.1 mmHg at 12 months, and the number of antiglaucoma medications declined from 2.08 ± 1.23 to 0.14 ± 0.52. The complete and qualified success rates were 80.6% and 88.3%, respectively. Postoperative complications occurred in 49 (17.0%) patients; while 3 (1.0%) patients underwent yttrium-aluminum-garnet (YAG) laser capsulotomy and 6 (2.1%) required glaucoma surgery. Multivariate Cox regression analysis revealed that the anterior chamber depth (*P* = 0.044) and axial length (*P* = 0.050) were positively associated with qualified success. The complete and qualified success rates among patients with advanced PACG were 78.2% and 87.9%, respectively.

**Conclusion:**

We demonstrated that PEI-GSL is an effective and safe treatment for patients with PACG and coexisting cataracts, even in advanced stages.

**Supplementary Information:**

The online version contains supplementary material available at 10.1186/s40662-026-00507-y.

## Background

Glaucoma is a leading cause of irreversible blindness worldwide. In 2020, it affected approximately 76 million individuals globally, and projections indicate that it will reach 111.8 million by 2040 [1]. Among the major subtypes, primary angle-closure glaucoma (PACG) affects approximately 23.4 million individuals worldwide [1], and nearly half live in China [2]. The risk of blindness associated with PACG is about three-fold higher than that of primary open-angle glaucoma [3]. Globally, PACG has blinded approximately 5.25 million individuals [2] and thus posed a substantial socioeconomic burden on families and societies.

Surgical intervention is crucial to prevent blindness in patients undergoing PACG. The surgical outcomes of conventional procedures such as trabeculectomy and phacotrabeculectomy have been good for PACG with cataracts [4–6]. However, they are frequently accompanied by complications such as hypotony, shallow anterior chamber, and bleb-related issues that significantly affect the quality of life of patients [7, 8]. A combination of phacoemulsification (PEI), goniosynechialysis (GSL), and goniotomy (GT) to manage PACG is potentially effective and safe [9, 10]. For example, Dorairaj et al. reported that 6- and 12-month cumulative success rates of treating PACG with cataracts using PEI + GSL + GT were 92.9% and 95.2%, respectively, without severe complications [10, 11]. However, adding GT might be associated with a higher rate of postoperative complications [12, 13], and the additional benefit compared with PEI - GSL to treat PACG remains unknown. Although the effectiveness of PEI-GSL for treating PACG has been documented, the studies were limited by small samples, early disease, or retrospective designs [14–18]. For example, Husain et al. indicated that the effects of PEI and PEI-GSL were comparable; however, patients with advanced PACG were excluded [16]. Furthermore, only two retrospective studies have investigated PEI-GSL in advanced PACG [18, 19]. Consequently, large-scale multicenter prospective cohort studies are urgently needed to comprehensively assess the real-world clinical outcomes of PEI-GSL.

Therefore, we conducted a large-scale, multicenter, prospective cohort study to thoroughly investigate the effectiveness and safety of PEI-GSL among patients with PACG accompanied by cataracts. We also analyzed potential factors associated with successful surgical outcomes.

## Methods

### Study design and participants

This multicenter prospective cohort study included consecutively enrolled patients with PACG and coexisting cataracts who underwent PEI-GSL between November 1, 2022, and March 12, 2024 [20]. The patients were recruited from the Eye Hospital of Wenzhou Medical University, Eye Hospital of Nanchang University, Ningbo Eye Hospital, Dalian Third People’s Hospital, and Tianjin Medical University Eye Hospital. The Ethics Committee at each Eye Hospital approved this study (Approval No: 2022-163-K-128-01, 2023-002-01, 2023-04(K)-C2, 2023-003-001, 2023KY-09), which complied with the ethical principles enshrined in the Declaration of Helsinki (2024 amendment) and by the ethics committees of the other participating hospitals. All the patients signed informed consent forms before participating in the study.

We defined PACG as ≥ 180° posterior trabecular meshwork that is invisible by gonioscopy, accompanied by glaucomatous optic neuropathy and an associated visual field defect [21, 22]. The exclusion criteria were as follows: (1) secondary angle-closure glaucoma; (2) previous glaucoma surgeries, such as trabeculectomy and glaucoma drainage device implantation; (3) previous ocular trauma or intraocular surgeries, such as vitreous surgeries and cataract extraction; and (4) active ocular inflammation, such as ocular infections and uveitis. Considering the potential influence of glaucoma severity on surgical outcomes, PACG was staged based on the mean deviation (MD) of the visual field [23]. Early-to-moderate and advanced PACG were defined as MD ≥ −12 and < −12 dB, respectively.

### Ocular examination

All patients underwent comprehensive ocular evaluations at baseline and were followed up 1 week later, then at 1, 3, 6, and 12 months postoperatively. A qualified ophthalmologist assessed the anterior and posterior segments using a slit-lamp biomicroscope (BQ900, Haag-Streit AG, Köniz, Switzerland). Visual acuity (VA) and best-corrected VA (BCVA) were measured using Snellen charts and converted into logarithms of the minimum angle of resolution (logMAR). Axial length (AL) and central anterior chamber depth (CACD) were respectively measured using an IOL Master (Carl Zeiss Meditec AG, Jena, Germany) and an ultrasound biomicroscope (Quantel Medical, Cournon d'Auvergne, France). The central corneal thickness (CCT) and endothelial cell density were assessed using a corneal endothelial microscope (CEM-530, NIDEK Co., Ltd., Gamagori, Japan). Visual fields were tested using a Humphrey Visual Field Analyzer (Carl Zeiss Meditec) following the SITA 24–2 standard protocol. Intraocular pressure (IOP) was measured using an AT 900 model T Goldmann applanation tonometer (Haag-Streit AG). Glaucoma specialists in a dark environment used a gonioscope with a Goldmann 2-mirror lens (Ocular O2M, Ocular Instruments Inc., Bellevue, WA, USA). Peripheral anterior synechiae (PAS) were considered if the iris–trabecular contact persisted for at least one-half clock hour during indentation gonioscopy.

### Surgical procedure and postoperative management

A senior ophthalmologist conducted all surgeries. Pupils were dilated 30 min before surgery. Clear corneal and auxiliary incisions of 2.2–3.0 and 1.0 mm were cut, and the anterior chamber was then filled with a viscoelastic agent, followed by continuous curvilinear capsulorhexis. After hydrodissection and hydrodelineation, the lens nucleus and cortex were removed using PEI. An intraocular lens (IOL) was implanted into the capsular bag. The extent of PAS was intraoperatively assessed by gonioscopy. Viscoelastic and iris repositors were used to separate PAS adhesions until the trabecular meshwork was fully visualized. A few patients required an additional auxiliary incision to ensure complete separation and adherent PAS. Residual viscoelastic material was removed from the anterior chamber, and all surgical steps were systematically documented.

Postoperative care included topical corticosteroid eye drops (1% tobramycin-dexamethasone) four times daily for two weeks, nonsteroidal anti-inflammatory drugs four times daily for one month, and topical corticosteroid ointment (tobramycin-dexamethasone) once a night for one week.

### Outcome measures

The primary outcomes were the IOP and the number of antiglaucoma medications administered 12 months postoperatively. Secondary outcomes included the cumulative success rate, incidence of complications, and reoperations. Complete success was defined as an IOP of 6–18 mmHg, without the use of antiglaucoma medications, and without the need for reoperation [13, 24]; and qualified success was defined as an IOP of 6–18 mmHg with or without antiglaucoma medications and no reoperation. A transient IOP spike was defined as a postoperative IOP ≥ 30 mmHg at any time. Hyphema was defined as blood stratification in the anterior chamber [25]. Reoperation was defined as any additional intervention in an operating room, including trabeculectomy, GSL, GT, and cyclophotocoagulation.

### Statistical analysis

Continuous variables were expressed as means ± standard deviation (SD), while categorical variables were presented as frequencies and percentages. Continuous variables were compared using a paired t-test or an independent sample t-test, and categorical variables were compared using the chi-square test. Complete and qualified success rates were assessed using Kaplan–Meier survival curves generated during follow-ups. Hazard ratios (HR) were calculated using Cox regression models, and 95% confidence intervals (CI) were calculated for factors associated with complete qualified success. Variables with *P* < 0.20 in a univariate analysis were included in subsequent multivariate models. All data were statistically analyzed using Stata v. 18.0 (Stata Corporation, College Station, TX, USA). Differences were considered statistically significant at a two-sided *P* < 0.05.

## Results

### Baseline characteristics of the participants

Among the enrolled patients, 237 (82.0%) of 289 completed a mean follow-up duration of 12.4 ± 1.8 months. Table 1 shows the baseline demographic and clinical characteristics of the patients. The mean age was 67.5 ± 8.7 years, and 166 (57.4%) patients were female, among whom 37 (12.8%) participants had undergone a laser peripheral iridotomy (LPI). Their BCVA was 0.48 ± 0.65 logMAR; mean deviation (MD), − 19.89 ± 10.45 dB; mean AL, 22.6 ± 0.84 mm; mean CACD, 1.89 ± 0.26 mm. Additional file 1 Table S1 shows the baseline characteristics of the patients who completed or were lost to follow-up. These groups were not significantly different.

**Table 1:** Demographics and clinical baselines of patients

Characteristics	Studied eyes
Number of eyes studied	289
Age (years)	67.5 ± 8.7
Female, n (%)	166 (57.4%)
Systolic pressure (mmHg)	134.6 ± 19.0
Diastolic pressure (mmHg)	78.0 ± 10.6
Hypertension, n (%)	117 (40.5%)
Diabetes, n (%)	52 (18.0%)
BCVA (logMAR)	0.48 ± 0.65
Previous LPI, n (%)	37 (12.8%)
Baseline IOP (mmHg)	22.9 ± 10.2
MD of visual field (dB)	− 19.89 ± 10.45
Mean PAS (°)	229.3 ± 109.8
Mean AL (mm)	22.6 ± 0.84
Mean CACD (mm)	1.89 ± 0.26
Mean CCT (μm)	529.9 ± 37.3

*AL =* axial length; *BCVA =* best-corrected visual acuity; *CACD =* central anterior chamber depth; *CCT =* central corneal thickness; *IOP =* intraocular pressure; *LPI =* laser peripheral iridotomy; *MD =* mean deviation; *PAS =* peripheral anterior synechiae; *SD =* standard deviation

Continuous data are presented as means with SD. Categorical data are shown as n (%)

### IOP and number of antiglaucoma medications

**Fig. 1:**
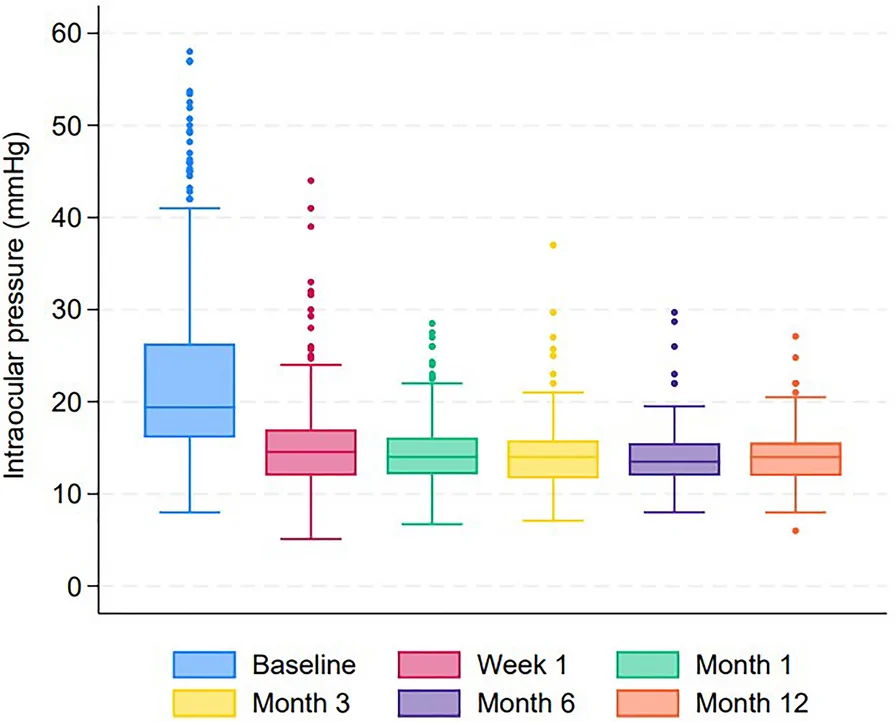
Intraocular pressure at baseline and at each follow-up point among patients with primary angle-closure glaucoma and coexisting cataracts

Table 2 lists the IOP of the patients at baseline and at each follow-up visit. The mean IOP at baseline decreased from 22.9 ± 10.2 to 14.0 ± 3.1 mmHg at 12 months postoperatively. The absolute reduction in IOP at 12 months was 8.8 ± 10.1 mmHg, corresponding to a 29.3% ± 27.1% decrease. The mean IOP at each follow-up point was significantly lower than the baseline value (*P* < 0.001). Figure 1 illustrates the IOP trend from baseline to each follow-up point. The mean number of antiglaucoma medications at baseline decreased from 2.08 ± 1.23 to 0.14 ± 0.52 at 12 months, whereas the proportions of patients who did not use them increased from 14.2% to 90.3%. Table 3 details the distribution of antiglaucoma medications at baseline and at each follow-up point. Furthermore, the baseline BCVA decreased from 0.48 ± 0.65 to 0.34 ± 0.70 logMAR at 12 months.

**Table 2:** Intraocular pressure at baseline and at each follow-up point

Variables	Baseline (n = 289)	Week l (n = 287)	Month 1 (n = 259)	Month 3 (n = 238)	Month 6 (n = 231)	Month 12 (n = 237)
Intraocular pressure (mmHg)	22.9 ± 10.2	15.4 ± 5.2	14.8 ± 3.7	14.1 ± 3.7	13.8 ± 3.3	14.0 ± 3.1
Absolute change from baseline (mmHg)	-	7.3 ± 11.1	8.1 ± 10.2	8.5 ± 10.6	8.6 ± 9.8	8.8 ± 10.1
Percentage change from baseline (%)	-	21.7 ± 38.1	25.9 ± 29.0	28.2 ± 29.4	30.0 ± 27.3	29.3 ± 27.1
P*	-	*P* < 0.001	*P* < 0.001	*P* < 0.001	*P* < 0.001	*P* < 0.001

^*^P < 0.001 indicates significant difference between each follow-up point and baseline IOP

**Table 3:** Number of antiglaucoma medications at baseline and at each follow-up point

No. of antiglaucoma medications	0	1	2	3	4	5
Baseline (n = 289)	41 (14.2)	43 (14.9)	92 (31.8)	81 (28.0)	28 (9.7)	4 (1.4)
Week 1 (n = 287)	221 (77.0)	34 (11.9)	16 (5.6)	14(4.9)	2 (0.7)	0 (0)
Month 1 (n = 259)	212 (81.9)	30 (11.6)	11 (4.3)	3 (1.2)	3 (1.2)	0 (0)
Month 3 (n = 238)	204 (85.7)	18 (7.6)	12 (5.0)	2 (0.8)	2 (0.8)	0 (0)
Month 6 (n = 231)	207 (89.6)	18 (7.8)	3 (1.3)	3 (1.3)	0 (0)	0 (0)
Month 12 (n = 237)	214 (90.3)	17 (7.2)	2 (0.8)	3 (1.3)	1 (0.4)	0 (0)

Data are presented as n (%). n represents the number of patients

### Cumulative success rate, complications, and reoperations

**Fig. 2:**
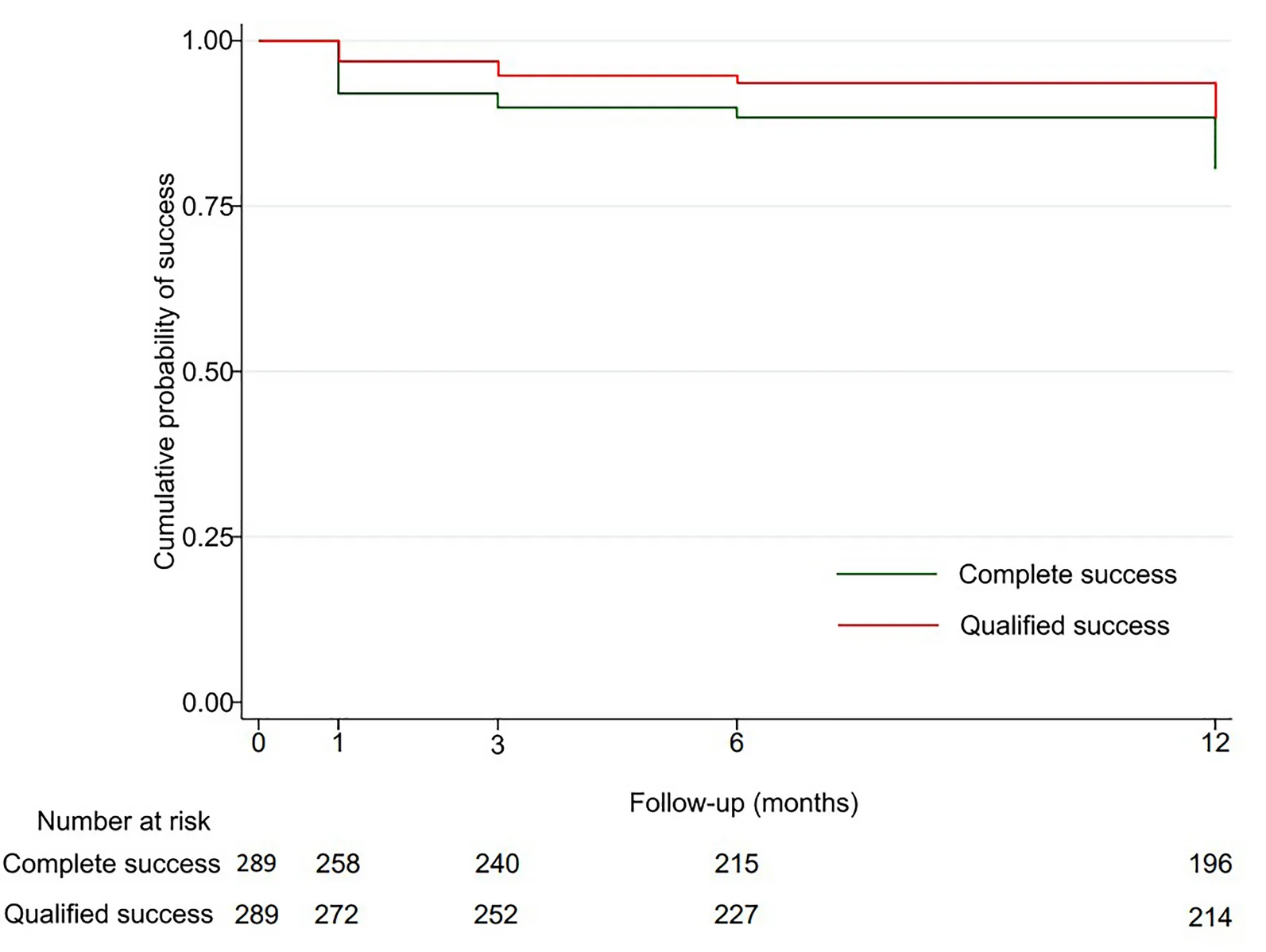
Kaplan–Meier survival curves showing cumulative surgical success during the follow-up after combined phacoemulsification and goniosynechialysis surgery

The Kaplan–Meier survival curves in Fig. 2 show the cumulative probability of success after PEI-GSL surgery. The complete and qualified success rates were 80.6% and 88.3%, respectively, during follow-up (IOP range, 6–18 mmHg). Furthermore, the complete and qualified success rates were 85.5% and 94.8%, respectively, when surgical success was defined as an IOP of 6–21 mmHg (Additional File 1 Figure S1).

We analyzed two subgroups based on preoperative IOP and the number of antiglaucoma medications. The complete (79.6% vs. 81.3%; *P* = 0.675) and qualified (86.5% vs. 89.5%; *P* = 0.420) success rates did not significantly differ between patients with preoperative IOP > 21 and ≤ 21 mmHg (Additional File 1 Figure S2). The complete (83.4% vs. 78.6%; *P* = 0.347) and qualified (90.7% vs. 86.5%; *P* = 0.299) success rates also did not significantly differ between patients with > 2 or ≤ 2 antiglaucoma medications (Additional File 1 Figure S3).

Table 4 summarizes postoperative complications and reoperations. A total of 56 (19.4%) postoperative complications occurred in 49 (17.0%) patients. The most prevalent complication was corneal edema (n = 27, 9.3%), followed by IOP spikes (n = 16, 5.5%), hyphema (n = 6, 2.1%), malignant glaucoma (n = 3, 1.0%), shallow anterior chamber (n = 3, 1.0%), and choroidal detachment (n = 1, 0.3%). All patients with IOP spikes experienced a return to normal range after using antiglaucoma medications. Three patients (1.0%) underwent yttrium-aluminum-garnet (YAG) laser posterior capsulotomy because of malignant glaucoma or a shallow anterior chamber. Six patients (2.1%) required reoperation for uncontrolled IOP or malignant glaucoma, including one with a prior YAG laser posterior capsulotomy. The reoperation procedures consisted of Kahook Dual Blade GT on postoperative day 4 (n = 1, 0.3%), cyclophotocoagulation at 1 week and 3 months postoperatively (n = 2, 0.7%), GSL at 1 and 3 months postoperatively (n = 2, 0.7%), and penetrating canaloplasty at 12 months postoperatively (n = 1, 0.3%).

**Table 4:** Complications and reinterventions after combined phacoemulsification and goniosynechialysis

Complications	All	Early to moderate PACG (n = 80)	Advanced PACG (n = 209)	*P*
Corneal edema	27 (9.3)	6 (7.5)	21 (10.1)	0.505
Hyphema	6 (2.1)	0 (0)	6 (2.9)	0.126
IOP spike	16 (5.5)	3 (3.8)	13 (6.3)	0.411
Shallow anterior chamber	3 (1.0)	0 (0)	3 (1.4)	0.281
Malignant glaucoma	3 (1.0)	0 (0)	3 (1.4)	0.281
Choroidal detachment	1 (0.3)	1 (1.3)	0 (0)	0.105
Total number of complications	56 (19.4)	10 (12.5)	46 (22.0)	-
Total patient of complications	49 (17.0)	9 (11.3)	40 (19.1)	0.110
Reinterventions				
YAG laser posterior Capsulotomy*	3 (1.0)	0 (0)	3 (1.4)	0.281
Reoperation	6 (2.1)	0 (0)	6 (2.9)	0.126
Total number of reinterventions	9 (3.1)	0 (0)	9 (4.3)	-
Total patients of reinterventions	8 (2.8)	0 (0)	8 (3.8)	0.076

*IOP =* intraocular pressure; *PACG =* primary angle-closure glaucoma; *YAG =* yttrium-aluminum-garnet

Data are presented as n (%)

^*^This postoperative procedure manages glaucoma problems

### Factors associated with surgical success

Additional file 1 Table S2 shows the results of the Cox regression analysis of potential factors associated with complete success. In the univariate Cox model, CACD (HR = 0.42; 95% CI: 0.12–0.85; *P* = 0.023) was significantly associated with complete success, but not in the multivariate model (*P* = 0.072). Age (HR = 0.95; 95% CI: 0.92–0.99; *P* = 0.016), CACD (HR = 0.29; 95% CI: 0.10–0.94; *P* = 0.039), and AL (HR = 0.59; 95% CI: 0.40–0.88; *P* = 0.010) significantly reduced the risk of failure for qualified success in the univariate Cox model (Additional file 1 Table S3), and CACD (HR = 0.25; 95% CI: 0.10–0.96; *P* = 0.044) reduced risk in the multivariate Cox model, whereas AL (HR = 0.67; 95% CI 0.45–1.00; *P* = 0.050) was marginally associated.

### Early-to-moderate and advanced PACG

The patients were assigned to groups based on the MD of their visual fields, namely, early-to-moderate (n = 80; Group 1) and advanced (n = 209; Group 2) PACG. Additional file 1 Figure S4 shows the IOP at baseline and the follow-ups for both groups. The mean IOP decreased from baseline values of 19.7 ± 8.4 and 23.9 ± 10.6 mmHg in Groups 1 and 2 to 14.0 ± 2.6 and 14.0 ± 3.3 mmHg at 12 months, respectively. The proportion of patients who did not use antiglaucoma medications increased from 27.5% to 97.1% and 9.1% to 87.4% in Groups 1 and 2, respectively (Additional file 1 Table S4). The Kaplan–Meier survival curves of Groups 1 and 2 show complete success and qualified rates of 86.7% and 78.2% (*P* = 0.084; Additional file 1 Figure S5) and 89.3% and 87.9%, respectively (*P* = 0.654). The incidence of postoperative corneal edema, IOP spike, hyphema, shallow anterior chamber, choroidal detachment, and malignant glaucoma did not significantly differ between the groups (*P* > 0.05; Table 4). Rates of YAG laser posterior capsulotomy (*P* = 0.281) and reoperation (*P* = 0.126) were also not significantly different.

## Discussion

We determined the effectiveness of PEI-GSL for patients with PACG accompanied by cataracts in this multicenter prospective study. The results showed that PEI-GSL significantly lowered the IOP and reduced the need for antiglaucoma medications. At 12 months postoperatively, the complete and qualified success rates were 80.6% and 88.3%, respectively. The outcomes did not significantly differ between patients with early-to-moderate and those with advanced PACG. The incidences of postoperative complications and reoperations were relatively low.

A randomized controlled trial that included 38 patients with PACG identified complete (IOP < 21 mmHg and decrease of 20%) and qualified success (IOP < 21 mmHg) rates of 57.9% and 92.1% in the PEI-GSL group at the 12-month follow-up, and IOP decreased by 27.8%, from a baseline of 22.9 ± 5.3 to 15.9 ± 4.5 mmHg [16]. Another study of 40 patients with PACG found that IOP decreased from a baseline of 30.72 ± 3.88 to 13.21 ± 1.97 mmHg at 6 months, and only 3 (7.5%) patients developed complications [26]. Furthermore, a retrospective study found qualified success rates of 85.9% and 66.2% for IOP < 21 and < 18 mmHg at 12 months postoperatively among 109 patients with PAC/PACG, respectively [17]. Our results showed that IOP decreased from a baseline of 22.9 ± 10.2 to 14.0 ± 3.1 mmHg at 12 months of follow-up, and the complete and qualified success rates were 80.6% and 88.3%, respectively. The present findings of a larger sample in a multicenter prospective study strengthened these findings. Moreover, the outcomes of our patients were better than those reported in previous retrospective studies, possibly due to the influence of selection bias, as patients with well-controlled IOP might have been lost to follow-up.

Phacotrabeculectomy can treat advanced PACG with cataracts [6, 27, 28], and it is a recommended treatment option [29], although it is associated with a high risk of postoperative complications [30]. Recent studies have shown that PEI + GSL + GT is safe and effective in advanced PACG with cataracts [10, 31]. For example, a randomized controlled trial found comparable efficacy between PEI + GSL + GT and phacotrabeculectomy, with qualified success rates of 90.8% and 93.2%, respectively, fewer postoperative complications and interventions, and a better quality of life [9]. We also found that PEI-GSL was safe and effective for treating advanced PACG. Furthermore, a case series of 18 eyes indicated that PEI combined with endoscopic GSL can effectively treat advanced PACG, with a success rate of 83.3% [18]. A multicenter randomized controlled trial is warranted to compare PEI + GSL + GT or phacotrabeculectomy in patients with advanced PACG.

Our finding that greater CACD and AL were associated with success rates likely reflects underlying anatomical advantages. A deeper anterior chamber reduces the risk of postoperative angle re-closure, and PEI-GSL enhances aqueous humor outflow [14]. A larger potential space between the posterior iris surface and the trabecular meshwork in eyes with deeper CACD facilitates sustained postoperative angle opening. Additionally, a longer AL is often associated with a more posteriorly positioned lens [29], which reduces the risk of postoperative pupillary block from anterior lens displacement. These anatomical factors collectively contribute to a more stable aqueous outflow and explain the favorable outcomes found in our patients.

Our study found that postoperative complications were relatively low risk when PACG was treated using PEI-GSL. This finding was consistent with previous reports [14, 16]. Specifically, hyphema occurred in only 2.1% of patients. An IOP spike was identified in 5.5% of our patients, compared with none in the PEI-GSL cohort of Husain et al. [16], but it was conspicuously lower than the 27.0% found in a retrospective study [19]. Malignant glaucoma occurred in 1.0% of our patients, the rate of which (0.75%) was similar to that reported for glaucoma surgery in patients with PACG [32], whereas such results of PEI-GSL have not been reported. Only 2.1% of our patients required reoperations due to uncontrolled IOP or malignant glaucoma.

This study has several strengths. This multicenter prospective design reflects the outcomes of PEI-GSL in terms of treating PACG in clinical practice. A large sample added robustness to the findings, particularly in the effectiveness and safety of PEI-GSL to treat advanced PACG. This provides further clinical evidence to support broader adoption. However, this study also has some limitations. This was a noncomparative observational study that did not directly compare PEI-GSL with other surgical interventions; future randomized controlled trials are needed to compare surgical approaches. A one-year follow-up was insufficient, but we are still following up our participants to observe long-term outcomes. Although further exploring the surgical outcomes at different PACG severity levels, the present sample size may be insufficient to detect clinical differences between groups. Although senior surgeons performed all procedures, variations in their expertise and differences in equipment among centers might have impacted the outcomes.

## Conclusions

In conclusion, this large multicenter prospective study confirmed that PEI-GSL significantly reduced both IOP and the need for antiglaucoma medications in patients with PACG and coexisting cataracts. We also found a relatively low incidence of postoperative complications, even among patients with advanced PACG. These findings further support PEI-GSL as an effective intervention for IOP control and a safe treatment option. Long-term outcomes and comparisons of randomized controlled trials with other surgical approaches are warranted.

## Supplementary Information

Supplementary Material 1

## Data Availability

The data that support the findings of this study are available from the corresponding author upon reasonable request.

## References

[1] Tham YC, Li X, Wong TY, Quigley HA, Aung T, Cheng CY. Global prevalence of glaucoma and projections of glaucoma burden through 2040: a systematic review and meta-analysis. Ophthalmology. 2014;121(11):2081–90. 24974815 10.1016/j.ophtha.2014.05.013

[2] Quigley HA, Broman AT. The number of people with glaucoma worldwide in 2010 and 2020. Br J Ophthalmol. 2006;90(3):262–7. 16488940 10.1136/bjo.2005.081224 PMC1856963

[3] George R, Panda S, Vijaya L. Blindness in glaucoma: primary open-angle glaucoma versus primary angle-closure glaucoma-a meta-analysis. Eye (Lond). 2022;36(11):2099–105. 34645961 10.1038/s41433-021-01802-9 PMC9582001

[4] Bindlish R, Condon GP, Schlosser JD, D’Antonio J, Lauer KB, Lehrer R. Efficacy and safety of mitomycin C in primary trabeculectomy: five-year follow-up. Ophthalmology. 2002;109(7):1336–41. 12093659 10.1016/s0161-6420(02)01069-2

[5] Tham CC, Kwong YY, Leung DY, Lam SW, Li FC, Chiu TY, et al. Phacoemulsification versus combined phacotrabeculectomy in medically controlled chronic angle closure glaucoma with cataract. Ophthalmology. 2008;115(12):2167–73.e2. 18801576 10.1016/j.ophtha.2008.06.016

[6] Tham CC, Kwong YY, Leung DY, Lam SW, Li FC, Chiu TY, et al. Phacoemulsification versus combined phacotrabeculectomy in medically uncontrolled chronic angle closure glaucoma with cataracts. Ophthalmology. 2009;116(4):725–31. 19243831 10.1016/j.ophtha.2008.12.054

[7] Rulli E, Biagioli E, Riva I, Gambirasio G, De Simone I, Floriani I, et al. Efficacy and safety of trabeculectomy vs. nonpenetrating surgical procedures: a systematic review and meta-analysis. JAMA Ophthalmol. 2013;131(12):1573–82. 24158640 10.1001/jamaophthalmol.2013.5059

[8] Gedde SJ, Heuer DK, Parrish RK 2nd, Tube Versus Trabeculectomy Study Group. Review of results from the Tube Versus Trabeculectomy Study. Curr Opin Ophthalmol. 2010;21(2):123–8. 20040872 10.1097/ICU.0b013e3283360b68 PMC5584063

[9] Song Y, Lin F, Lv A, Zhang Y, Lu L, Xie L, et al. Phacogoniotomy versus phacotrabeculectomy for advanced primary angle-closure glaucoma with cataract: a randomized non-inferiority trial. Asia Pac J Ophthalmol (Phila). 2024;13(1):100033. 38383075 10.1016/j.apjo.2023.100033

[10] Dorairaj S, Tam MD, Balasubramani GK. Two-year clinical outcomes of combined phacoemulsification, goniosynechialysis, and excisional goniotomy for angle-closure glaucoma. Asia Pac J Ophthalmol (Phila). 2020;10(2):183–7. 33031110 10.1097/APO.0000000000000321

[11] Dorairaj S, Tam MD. Kahook dual blade excisional goniotomy and goniosynechialysis combined with phacoemulsification for angle-closure glaucoma: 6-month results. J Glaucoma. 2019;28(7):643–6. 30950968 10.1097/IJG.0000000000001256

[12] Yang F, Ma Y, Liang Z, Lv K, Yang K, Wu H. Combined phacoemulsification, goniosynechialysis and ab interno trabeculectomy in primary angle-closure glaucoma: long-term results. Int J Med Sci. 2025;22(2):451–9. 39781536 10.7150/ijms.103795 PMC11704697

[13] Gupta S, Sethi A, Yadav S, Azmira K, Singh A, Gupta V. Safety and efficacy of incisional goniotomy as an adjunct with phacoemulsification in primary angle-closure glaucoma. J Cataract Refract Surg. 2021;47(4):504–11. 33181630 10.1097/j.jcrs.0000000000000481

[14] Zhang H, Tang G, Liu J. Effects of phacoemulsification combined with goniosynechialysis on primary angle-closure glaucoma. J Glaucoma. 2016;25(5):e499–503. 26066498 10.1097/IJG.0000000000000297

[15] Rodrigues IA, Alaghband P, Beltran Agullo L, Galvis E, Jones S, Husain R, et al. Aqueous outflow facility after phacoemulsification with or without goniosynechialysis in primary angle closure: a randomised controlled study. Br J Ophthalmol. 2017;101(7):879–85. 28400374 10.1136/bjophthalmol-2016-309556

[16] Husain R, Do T, Lai J, Kitnarong N, Nongpiur ME, Perera SA, et al. Efficacy of phacoemulsification alone vs. phacoemulsification with goniosynechialysis in patients with primary angle-closure disease: a randomized clinical trial. JAMA Ophthalmol. 2019;137(10):1107–13. 31294768 10.1001/jamaophthalmol.2019.2493 PMC6624811

[17] Kameda T, Inoue T, Inatani M, Tanihara H, Japanese Phaco-Goniosynechialysis Multicenter Study Group. Long-term efficacy of goniosynechialysis combined with phacoemulsification for primary angle closure. Graefes Arch Clin Exp Ophthalmol. 2013;251(3):825–30. 22743828 10.1007/s00417-012-2091-8

[18] Qian Z, Huang J, Song B, Wei L, Fu L, Austin MW, et al. Cataract surgery (phacoemulsification with intraocular lens implantation) combined with endoscopic goniosynechialysis for advanced primary angle-closure glaucoma. Ophthalmol Glaucoma. 2021;4(4):365–72. 33242682 10.1016/j.ogla.2020.11.003

[19] Qian Z, Pan W, Nie L, Lin L, Wei L. Efficacy and safety of phaco-goniosynechialysis in advanced primary angle closure glaucoma with severe visual field loss. J Glaucoma. 2024;33(11):900–7. 39093019 10.1097/IJG.0000000000002474

[20] Xiong K, Wang Y, Yu B, Zhang S, Li G, Huang Q, et al. The extent of peripheral anterior synechiae and efficacy of combined phacoemulsification and goniosynechialysis in primary angle-closure glaucoma and cataracts: a multicenter prospective study. Asia Pac J Ophthalmol (Phila). 2026;15(1):100279. 41525893 10.1016/j.apjo.2026.100279

[21] Gedde SJ, Chen PP, Muir KW, Vinod K, Lind JT, Wright MM, et al. Primary Angle-Closure Disease Preferred Practice Pattern®. Ophthalmology. 2021;128(1):P30–70. 34933744 10.1016/j.ophtha.2020.10.021

[22] European Glaucoma Society Terminology and Guidelines for Glaucoma, 5th Edition. Br J Ophthalmol. 2021;105(Suppl 1):1–169. 10.1136/bjophthalmol-2021-egsguidelines 34675001

[23] Brusini P, Johnson CA. Staging functional damage in glaucoma: review of different classification methods. Surv Ophthalmol. 2007;52(2):156–79. 17355855 10.1016/j.survophthal.2006.12.008

[24] Chen DZ, Sng CCA, Sangtam T, Thomas A, Shen L, Huang PK, et al. Phacoemulsification vs. phacoemulsification with micro-bypass stent implantation in primary angle closure and primary angle closure glaucoma: a randomized single-masked clinical study. Clin Exp Ophthalmol. 2020;48(4):450–61. 32003538 10.1111/ceo.13721

[25] Shinmei Y, Kijima R, Nitta T, Ishijima K, Ohguchi T, Chin S, et al. Modified 360-degree suture trabeculotomy combined with phacoemulsification and intraocular lens implantation for glaucoma and coexisting cataract. J Cataract Refract Surg. 2016;42(11):1634–41. 27956291 10.1016/j.jcrs.2016.08.016

[26] Angmo D, Shakrawal J, Gupta B, Yadav S, Pandey RM, Dada T. Comparative evaluation of phacoemulsification alone versus phacoemulsification with goniosynechialysis in primary angle-closure glaucoma: a randomized controlled trial. Ophthalmol Glaucoma. 2019;2(5):346–56. 32672677 10.1016/j.ogla.2019.05.004

[27] Tsai HY, Liu CJ, Cheng CY. Combined trabeculectomy and cataract extraction versus trabeculectomy alone in primary angle-closure glaucoma. Br J Ophthalmol. 2009;93(7):943–8. 19383599 10.1136/bjo.2008.151803

[28] Elwehidy AS, Bayoumi NHL, Badawi AE, Hagras SM, Kamel R. Combined phacoemulsification-viscosynechialysis-trabeculotomy vs. phacotrabeculectomy in uncontrolled primary angle-closure glaucoma with cataract. J Cataract Refract Surg. 2019;45(12):1738–45. 31856984 10.1016/j.jcrs.2019.07.031

[29] Vass C, Menapace R. Surgical strategies in patients with combined cataract and glaucoma. Curr Opin Ophthalmol. 2004;15(1):61–6. 14743022 10.1097/00055735-200402000-00012

[30] Tham CC, Kwong YY, Leung DY, Lam SW, Li FC, Chiu TY, et al. Phacoemulsification vs. phacotrabeculectomy in chronic angle-closure glaucoma with cataract: complications [corrected]. Arch Ophthalmol. 2010;128(3):303–11. 20212200 10.1001/archophthalmol.2010.12

[31] Song Y, Xie L, Zhu X, Fan S, Lv A, Tang G, et al. Two-year outcome of phacogoniotomy for advanced primary angle-closure glaucoma with cataracts: a multicentre study. Br J Ophthalmol. 2025;109(7):770–4. 40049755 10.1136/bjo-2024-325375

[32] Lin H, Li J, Zheng X, Wan R, Zhou M, Ding Y, et al. Incidence and characteristics of aqueous misdirection after glaucoma surgery in Chinese patients with primary angle-closure glaucoma. Eye Vis (Lond). 2023;10(1):28. 37391810 10.1186/s40662-023-00346-1 PMC10314601

