# Supplementary material for: One-year outcomes of combined phacoemulsification and goniosynechialysis in primary angle-closure glaucoma with cataracts: a multicenter prospective study
Source: Eye Vis (Lond). 2026 Aug 20;13:36. doi: 10.1186/s40662-026-00507-y (PMC13491938; doi:10.1186/s40662-026-00507-y)
Supplement: Supplementary file 1 [file 40662_2026_507_MOESM1_ESM.docx]

**Additional file 1**

**Figure S1.** Kaplan–Meier survival curves during follow-up after combined phacoemulsification and goniosynechialysis surgery (6 ≤ IOP ≤ 21 mmHg).

**Figure S2.** Kaplan–Meier survival curves of subgroup based on preoperative intraocular pressure (IOP).

**Figure S3.** Kaplan–Meier survival curves of subgroups according to numbers of antiglaucoma medications.

**Figure S4.** Intraocular pressure at baseline and each of 6 follow-up points in patients with early-to-moderate and advanced primary angle-closure glaucoma (PACG).

**Figure S5.** Kaplan–Meier survival curves during follow-up after combined phacoemulsification and goniosynechialysis in patients with early-to-moderate and advanced primary angle-closure glaucoma (PACG).

**Table S1.** Comparisons of demographics and clinical baselines of patients who completed follow-up and those who did not

| **Characteristics** | **Follow-up completed** | **Follow-up incomplete** | ***P*** |
| --- | --- | --- | --- |
| Number of eyes | 237 | 52 | - |
| Age (years) | 67.1 ± 8.5 | 69.4 ± 9.6 | 0.084 |
| Female, n (%) | 137 (57.8%) | 29 (55.8%) | 0.788 |
| Systolic pressure (mmHg) | 134.3 ± 19.2 | 135.9 ± 17.9 | 0.570 |
| Diastolic pressure (mmHg) | 77.7 ± 10.8 | 79.4 ± 9.3 | 0.296 |
| Hypertension, n (%) | 97 (40.9%) | 20 (38.5%) | 0.743 |
| Diabetes, n (%) | 45 (19.0%) | 7 (13.5%) | 0.348 |
| BCVA (LogMAR) | 0.47 ± 0.65 | 0.56 ± 0.63 | 0.358 |
| Previous LPI, n (%) | 34 (14.4%) | 3 (5.8%) | 0.094 |
| Baseline IOP (mmHg) | 22.9 ± 10.2 | 22.8 ± 21.8 | 0.502 |
| MD of visual field (dB) | −19.52 ± 10.54 | −21.59 ± 9.94 | 0.205 |
| Mean AL (mm) | 22.6 ± 0.87 | 22.8 ± 0.66 | 0.190 |
| Mean CACD (mm) | 1.90 ± 0.26 | 1.86 ± 0.26 | 0.355 |
| Mean CCT (μm) | 530.2 ± 38.1 | 528.4 ± 33.6 | 0.778 |

AL = axial length; BCVA = best-corrected visual acuity; CACD = central anterior chamber depth; CCT = central corneal thickness; IOP = intraocular pressure; LPI = laser peripheral iridotomy; MD = mean deviation

Continuous and categorical data are shown as means ± SD, or n (%)

**Table S2.** Results of the Cox regression analysis for predictors of complete success

| **Variables** | **Univariate** | | **Multivariate** | |
| --- | --- | --- | --- | --- |
|  | **HR (95% CI)** | ***P-value*** | **HR (95% CI)** | ***P-value*** |
| Age (per year) | 0.98 (0.95–1.01) | 0.124 | 0.98 (0.95–1.01) | 0.141 |
| Sex (male) | 0.90 (0.52–1.56) | 0.702 |  |  |
| Hypertension | 0.85 (0.48–1.49) | 0.562 |  |  |
| Diabetes | 1.20 (0.61–2.33) | 0.597 |  |  |
| Previous LPI | 0.83 (0.36–1.95) | 0.673 |  |  |
| Baseline IOP (per mmHg) | 1.01 (0.98–1.03) | 0.502 |  |  |
| MD of visual field (per dB) | 0.98 (0.95–1.00) | 0.098 | 0.98 (0.95–1.01) | 0.101 |
| Baseline extent of PAS | 0.99 (0.92–1.07) | 0.874 |  |  |
| CACD (per mm) | 0.42 (0.12–0.85) | 0.023 | 0.38 (0.13–1.09) | 0.072 |
| AL (per mm) | 0.73 (0.53–1.01) | 0.059 | 0.80 (0.58–1.11) | 0.183 |
| ECD (cells/mm^2^) | 1.00 (1.00–1.00) | 0.979 |  |  |

AL = axial length; CACD = central anterior chamber depth; ECD = endothelial cell density; HR = hazard ratios; IOP = intraocular pressure; LPI = laser peripheral iridotomy; MD = mean deviation; PAS = peripheral anterior synechiae **Table S3.** Results of the Cox regression analysis for predictors of qualified success

| **Variables** | **Univariate** | | **Multivariate** | |
| --- | --- | --- | --- | --- |
|  | **HR (95% CI)** | ***P*** | **HR (95% CI)** | ***P*** |
| Age (per year) | 0.95 (0.92–0.99) | 0.016 | 0.96 (0.92–1.00) | 0.056 |
| Sex (male) | 0.83 (0.40–1.71) | 0.612 |  |  |
| Hypertension | 0.80 (0.38–1.67) | 0.556 |  |  |
| Diabetes | 1.29 (0.55–2.99) | 0.556 |  |  |
| Previous LPI | 0.74 (0.30–1.83) | 0.509 |  |  |
| Baseline IOP (per mmHg) | 1.02 (0.99–1.05) | 0.188 | 1.01 (0.98–1.04) | 0.431 |
| MD of visual field (per dB) | 0.99 (0.95–1.02) | 0.460 |  |  |
| Baseline extent of PAS | 0.98 (0.87–1.08) | 0.635 |  |  |
| CACD (per mm) | 0.29 (0.10–0.94) | 0.039 | 0.25 (0.10–0.96) | 0.044 |
| AL (per mm) | 0.59 (0.40–0.88) | 0.010 | 0.67 (0.45–1.00) | 0.050 |
| ECD (cells/mm^2^) | 1.00 (1.00–1.00) | 0.320 |  |  |

AL = axial length; CACD = central anterior chamber depth; ECD = endothelial cell density; HR = hazard ratio; IOP = intraocular pressure; LPI = laser peripheral iridotomy; MD = mean deviation; PAS = peripheral anterior synechiae

**Table S4.** Numbers of antiglaucoma medications at baseline and five follow-up points based

on primary angle-closure glaucoma (PACG) severity

| **PACG severity** | **No. of antiglaucoma medications** | **0** | **1** | **2** | **3** | **4** | **5** |
| --- | --- | --- | --- | --- | --- | --- | --- |
| Early-to- moderate | Baseline (n = 80) | 22 (27.5) | 23 (28.8) | 14 (17.5) | 15 (18.8) | 6 (7.5) | 0 (0) |
|  | Month 12 (n = 70) | 68 (97.1) | 2 (2.9) | 0 (0) | 0 (0) | 0 (0) | 0 (0) |
| Advanced | Baseline (n = 209) | 19 (9.1) | 20 (9.6) | 78 (37.3) | 66 (31.6) | 22 (10.5) | 4 (1.9) |
|  | Month 12 (n = 167) | 146 (87.4) | 15 (9.0) | 2 (1.2) | 3 (1.8) | 1 (0.6) | 0 (0) |

Data are presented as n (%). n represents the number of patients.
